# Using Co-authorship Networks to Map and Analyse Global Neglected Tropical Disease Research with an Affiliation to Germany

**DOI:** 10.1371/journal.pntd.0004182

**Published:** 2015-12-31

**Authors:** Max Ernst Bender, Suzanne Edwards, Peter von Philipsborn, Fridolin Steinbeis, Thomas Keil, Peter Tinnemann

**Affiliations:** 1 Institute for Social Medicine, Epidemiology and Health Economics, Charité-Universitätsmedizin Berlin, Berlin, Germany; 2 Universities Allied for Essential Medicines Europe e.V. (UAEM), Berlin, Germany; 3 Department of Health Care Management, Berlin University of Technology, Berlin, Germany; 4 Faculty of Medicine, Technische Universität München, Munich, Germany; 5 Faculty of Medicine, Charité-Universitätsmedizin Berlin, Berlin, Germany; Duke University, UNITED STATES

## Abstract

**Background:**

Research on Neglected Tropical Diseases (NTDs) has increased in recent decades, and significant need-gaps in diagnostic and treatment tools remain. Analysing bibliometric data from published research is a powerful method for revealing research efforts, partnerships and expertise. We aim to identify and map NTD research networks in Germany and their partners abroad to enable an informed and transparent evaluation of German contributions to NTD research.

**Methodology/Principal Findings:**

A SCOPUS database search for articles with German author affiliations that were published between 2002 and 2012 was conducted for kinetoplastid and helminth diseases. Open-access tools were used for data cleaning and scientometrics (OpenRefine), geocoding (OpenStreetMaps) and to create (Table2Net), visualise and analyse co-authorship networks (Gephi). From 26,833 publications from around the world that addressed 11 diseases, we identified 1,187 (4.4%) with at least one German author affiliation, and we processed 972 publications for the five most published-about diseases. Of those, we extracted 4,007 individual authors and 863 research institutions to construct co-author networks. The majority of co-authors outside Germany were from high-income countries and Brazil. Collaborations with partners on the African continent remain scattered. NTD research within Germany was distributed among 220 research institutions. We identified strong performers on an individual level by using classic parameters (number of publications, h-index) and social network analysis parameters (betweenness centrality). The research network characteristics varied strongly between diseases.

**Conclusions/Significance:**

The share of NTD publications with German affiliations is approximately half of its share in other fields of medical research. This finding underlines the need to identify barriers and expand Germany’s otherwise strong research activities towards NTDs. A geospatial analysis of research collaborations with partners abroad can support decisions to strengthen research capacity, particularly in low- and middle-income countries, which were less involved in collaborations than high-income countries. Identifying knowledge hubs within individual researcher networks complements traditional scientometric indicators that are used to identify opportunities for collaboration. Using free tools to analyse research processes and output could facilitate data-driven health policies. Our findings contribute to the prioritisation of efforts in German NTD research at a time of impending local and global policy decisions.

## Author Summary

Neglected tropical disease research has changed considerably in recent decades, and the German government is committed to addressing its past neglect of NTD research. Our aim was to use an innovative social network analysis of bibliometric data to map neglected tropical disease research networks that are inside of and affiliated with Germany, thereby enabling data-driven health policy decision-making. We created and analysed co-author networks from publications in the SCOPUS database, with a focus on five diseases. We found that Germany's share of global publication output for NTDs is approximately half that of other medical research fields. Furthermore, we identified institutions with prominent NTD research within Germany and strong research collaborations between German institutions and partners abroad, mostly in other high-income countries. This allowed an assessment of strong collaborations for further development, e.g., for research capacity strengthening in low-income-countries, but also for identifying missed opportunities for collaboration within the network. Through co-authorship network analysis of individual researcher networks, we identified strong performers by using classic bibliometric parameters, and we identified academic talent by social network analysis parameters on an individual level.

## Introduction

In recent decades, global efforts against neglected tropical diseases (NTDs) have undoubtedly been successful in raising awareness and implementing ambitious treatment programs [1]. NTD research and development (R&D) efforts have shown a substantial increase [2], and necessary resources have largely been made available by the philanthropic and public sectors [3]. Although advances have been made, funding for global NTD R&D has been flatlining more recently, and significant need gaps in diagnostic and treatment tools prevail [4]. The global strain on public finances as well as questions about research inputs, e.g., regarding the coordination of international efforts, appropriate prioritisation and the return on investment (RoI), are leading to an increasing pressure to use limited resources most efficiently [5,6]. A Global Health R&D observatory has been proposed by the World Health Assembly to identify research needs to provide better information on where, by whom and what type of research is conducted, and to match limited resources with public health priorities more effective and efficiently [7].

With respect to R&D for NTDs, defining and measuring research output, research productivity and value in return for funding have received comparatively little attention. Furthermore, research processes and collaborations, such as scientific networks, have seldom been used for evidence-driven policy analysis. However, new tools and software are increasingly available to facilitate analysis in this field [8,9].

Bibliometric metadata of scientific publications are used to map and visualise scientific activity within countries or regions [10,11], and co-authorship network analysis is used to explore and quantify R&D collaboration between authors, institutions or countries [12].

In co-authorship networks, authors in the network are linked as nodes via co-authored scientific publications. These networks can be visualised as graphs in which each author represents a node in the network and each co-authored publication is represented by links, or edges, between the nodes. Measurements can describe the network structure, e.g., by density or centrality of authors. For an explanation and description of terminology used in social network analysis (SNA), see Table 1. In particular, a high betweenness centrality of individual author nodes indicates that they are connecting parts of a network that would only be poorly connected otherwise, or are not connected at all, and these nodes are interpreted as innovation hubs within networks [13,14].

**Table 1:** Glossary of network analysis terminology.

Term	Definition	Explanation
Node	Nodes represent actors within a network.	A node represents the individual authors (or research institutions) within the co-authorship networks.
Edge	Edges represent ties or relations within a network.	The edges in the network represent the co-authorship of different authors. All authors in the network that have published together in the covered timeframe are connected through an edge.
Betweenness Centrality	The betweenness centrality score is a measure of how often a node lies on the shortest path between nodes in the network[15]. Nodes with a high betweenness centrality often connect components of a network that would be disconnected if the node is removed.	A high betweenness centrality indicates that an author is frequently identified if you want to connect other authors in the co-authorship network with one another, and he/she lies "between" them as an intermediary.
Average degree	The degree states the quantity of direct neighbours of a node in a network.	Here, the degree states the sum of co-authors the respective author has published with in the covered timeframe. The average degree is calculated separately for each disease network.
(Giant) Component	Components of a graph are sub-graphs that are connected within but disconnected between sub-graphs. The term "giant component" is used for the sub-graph with the most nodes in the network [16].	Different components of the co-authorship network contain authors that are connected with one another through joint publications. They have not published with authors in the other components of the network within the covered timeframe and are therefore not connected in the network.
Graph density	Graph density is a measurement of how close the network is to being complete. If all nodes of a network are connected to each other, the graph density equals one [17].	For research networks, the graph density can be used as an indicator of how many possibilities there are for further collaborations between authors.

Along with other governments and non-governmental organisations, the German government acknowledges the fight to tackle NTDs as a continuing major global challenge and sees improved R&D efforts and capacity building as a way forward [18].

However, German global health and NTD research has been considered to be scarce in comparison with activities in comparable industrialized countries [19,20]. Other EU countries that usually show a similar or smaller amount of publication output in other research fields, such as the United Kingdom or France, have been shown to outperform Germany considerably in the field of NTD research [10]. As a late but committed entrant into this global effort, Germany can gain a lot by using tools effectively and efficiently to prioritize its resources and capabilities.

By using innovative tools such as network analysis, we aimed to both demonstrate the potential of this tool and to identify and map existing NTD research outputs and processes within Germany and with its partners abroad to enable informed, evidence-driven policy making that addresses the needs of patients with NTDs.

## Methods

### Database and search strategy

**Fig 1:**
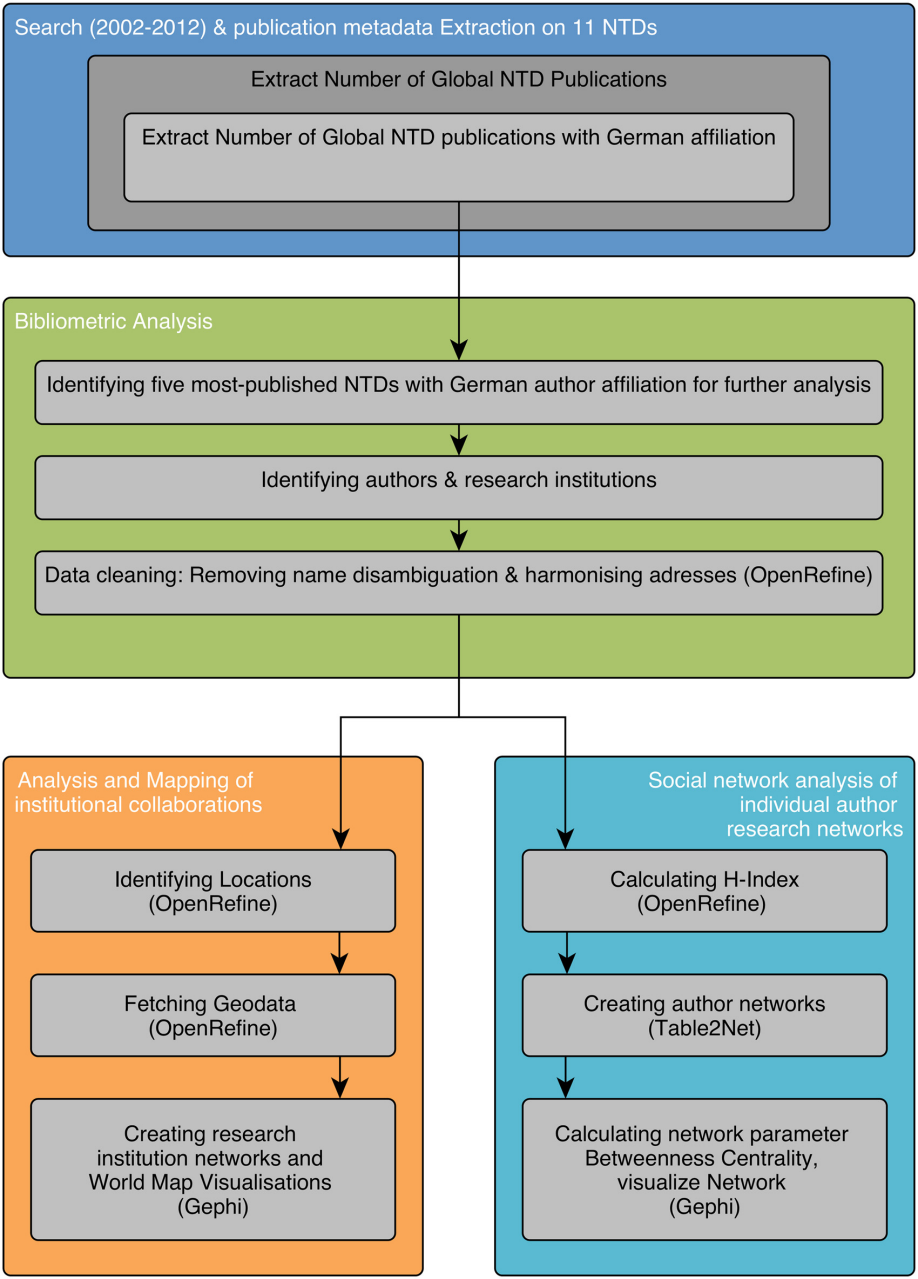
Flow chart, step-by-step methodology.

Initially, separate searches using the SCOPUS (www.scopus.com) database were performed for individual diseases to allow for the systematic identification of all relevant publications published between 2002 and 2012 on 11 kinetoplastid and helminthic diseases, and the NTD groups receiving the highest German government research funding [21,22]. Their metadata was extracted to build co-authorship research networks (Fig 1).

The search string used here included the disease name or a combination of the scientific name and common name as found on the WHO Neglected Tropical Disease Website (http://www.who.int/neglected_diseases/diseases/en/). A list of diseases and the search string used for further analysis are listed in Table 2.

**Table 2:** List of NTDs in the scope with scientific and common names and the search string used for bibliometric searches.

Search String: ‘TITLE-ABS-KEY(scientific disease name) OR TITLE-ABS-KEY(common name) AND AFFIL(germany) AND PUBYEAR > 2001 AND PUBYEAR < 2013’	
Kinetoplastids	Helminths
Leishmaniasis	Onchocerciasis / River Blindness
Chagas / American Trypanosomiasis	Lymphatic Filariasis / Elephantiasis
Human African Trypanosomiasis / Sleeping Sickness	Ascariasis / Roundworm
	Hookworm Infection
	Cysticercosis / Taeniasis
	Trichuriasis / Whipworm
	Dracunculiasis / Guinea worm disease
	Schistosomiasis

No filtering was performed for the research type (e.g., basic, clinical, and operational research) to acknowledge the diversity of NTD research needs [23]. To allow a comparison between overall international and German research output, the searches were first performed without a location filter, and during a second step, an affiliation filter for Germany was applied. Only publications with at least one co-author affiliated with a German institution were included.

### Data cleaning, and identifying authors and research institutions

The results of the SCOPUS database search with the Germany affiliation filter were exported. We further analysed the datasets for the five diseases with the highest number of publications.

OpenRefine software (www.openrefine.org) was used for data cleaning for all exported datasets. The given clustering algorithms were used to remove duplicates and resolve name disambiguation issues [24] for both authors and research institutions. Additional data cleaning was conducted manually afterwards.

Bibliometric data provided by the SCOPUS database was used to calculate the h-index for individual authors in each set of publications for individual diseases to establish measures for individual author output productivity (number of publications) and impact (number of citations by other researchers) for further analysis. To create the h-index, citations for each author's publications were collected from the SCOPUS exports of each disease, and individual author h-indices were calculated specifically for each disease as defined by Hirsch [25].

For the spatial visualisation of the international research organisation networks, individual author affiliations from the bibliometric data were manually harmonised and geocoded using the OpenStreetMaps (www.openstreetmaps.org) application programing interface (API) within OpenRefine.

### Creating research networks for authors and institutions

By using the web-based Table2Net tool [26], the processed database was set up to create co-authorship networks [27]. Networks for each disease were created for both collaborating co-authors and collaborating research institutions using author names or research organisations that were named as affiliations to identify nodes within the network (S1–S10 Datasets) [28]. The title of the co-authored publication was used to identify edges connecting individual nodes in both sets of networks. If authors or institutions collaborated more than once, the edges were weighted by the number of collaborations between the same authors or institutions. The individual disease networks represent all authors or institutions with contributions to NTD research publications that have at least one co-author working at a German research institution.

The Gephi software was used to calculate the occurrence counts and the network analysis parameter betweenness centrality (see Table 1). Authors were then ranked by their occurrence count, h-index and betweenness centrality. For the visualisation of co-author networks, the ForceAtlas 2 layout was used, and the global positioning system (GPS) coordinates for research institutions were used to visualise institutional collaborations on a world map [29].

All software used to analyse the data exported from SCOPUS is open-source and available free of charge.

## Results

### International publication output comparison

**Fig 2:**
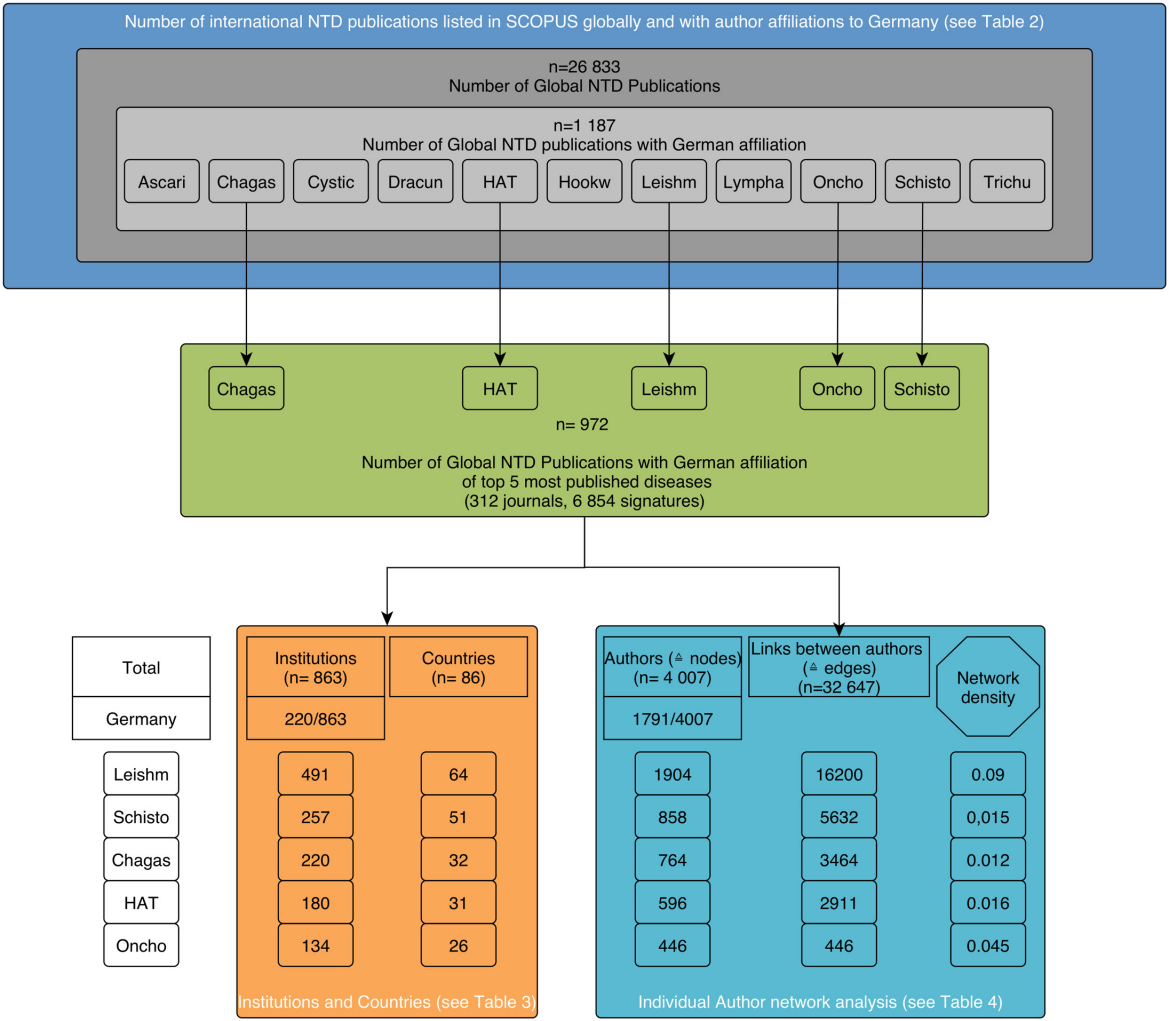
Flow chart, step-by-step results (with reference to results in Tables 3, 4 and 5).

An overview of the search results, bibliometric data and the following analysis of institutional collaborations and individual author research networks is presented in Fig 2.

Combined searches for publications on the 11 kinetoplastid and helminth NTDs revealed more than one thousand publications with at least one German author affiliation, representing 4.4% of the total number of publications published internationally about the different NTDs (Table 3).

**Table 3:** Number of international NTD publications listed in SCOPUS from around the world and with author affiliations to Germany by diseases, as ordered by the number of publications with German affiliations.

Disease	Number of international NTD publications (in %)	Number of international NTD publications with German affiliations (in %)
Leishmaniasis	8300 (30.9%)	407 (34.3%)
Schistosomiasis	5145 (19.2%)	164 (13.8%)
Chagas Disease	4951 (18.5%)	149 (12.6%)
Sleeping Sickness	1468 (5.5%)	130 (11.0%)
Onchocerciasis	840 (3.1%)	123 (10.4%)
Lymphatic Filariasis	1379 (5.1%)	65 (5.5%)
Ascariasis	1368 (5.1%)	50 (4.2%)
Hookworm Infection	1139 (4.2%)	41 (3.5%)
Cysticercosis	1510 (5.6%)	28 (2.4%)
Trichuriasis	595 (2.1%)	25 (2.1%)
Dracunculiasis	138 (0.5%)	5 (0.4%)
**Total (all diseases)**	**n = 26 833 (100%)**	**n = 1187 (100%)**

For the 11 diseases included in our analysis, international research efforts were predominantly related to three diseases, whereas the German NTD research efforts were more equally spread across five diseases. The three most researched NTDs internationally (Leishmaniasis, Chagas disease and Schistosomiasis) comprise 68.6% of the cumulative international publications, compared with 60.7% for those with German author affiliations. In contrast, for the top five most frequently researched NTDs (the top three diseases plus Sleeping Sickness and Onchocerciasis), the proportion was 77.2% internationally and 82.1% for those with German author affiliations. More than a fifth of the publications with German author affiliations were in the fields of Sleeping Sickness (11%) and Onchoceriasis (10%) compared with less than a tenth of international publications (Sleeping Sickness 5%; Onchocerciasis 3%).

### Co-authorship networks for the five most frequently published NTDs

**Fig 3:**
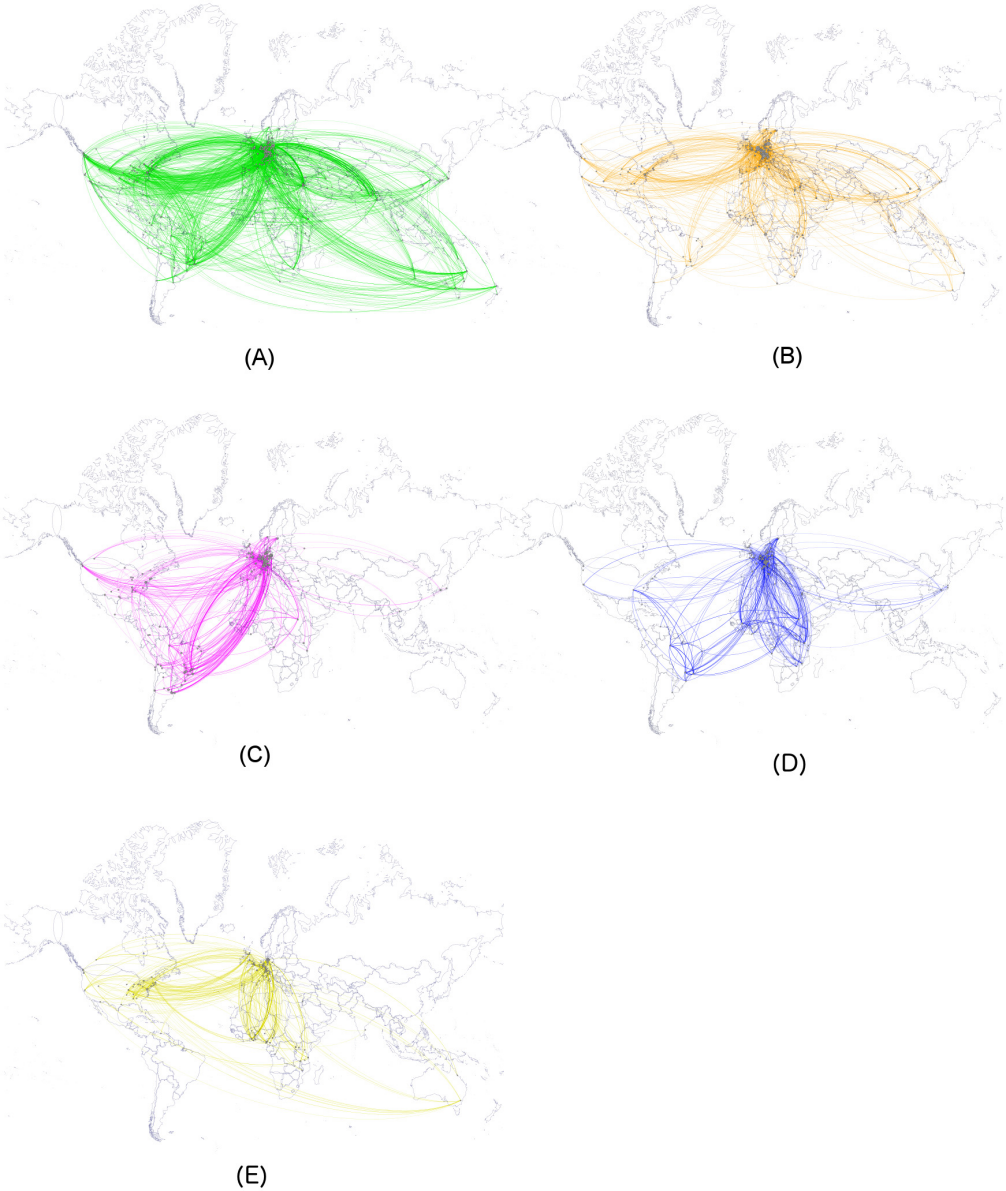
Worldwide connections between German research institutions and partners abroad. Research networks are based on co-author networks affiliated with Germany, i.e., those covering publications with at least one co-author affiliated with a German institution; nodes (circles) indicate research institutions, and edges (colored lines) indicate co-authored publications between authors based at those institutions. The maps show research networks for (A) Leishmaniasis, (B) Schistosomiasis, (C) Chagas disease, (D) Sleeping Sickness and (E) Onchocerciasis.

For the five diseases that formed the focus of our analysis (see Fig 3), we found a total of 972 publications with author affiliations from German institutions for 2002–2012. Of these, 908 publications were unique to one disease, and 64 publications appeared in two or more searches. Among the articles, 4711 authors and 3803 research institutions were identified. After duplicates were removed and name disambiguation was performed through computational and manual data cleaning, we identified 4568 authors and 1502 research institutions, but because some authors and research institutions were named in publications for more than one of the five diseases, we eventually identified 4007 individual authors and 863 research institutions.

Of the 972 publications, 96.5% were published in collaboration with two or more authors; 6,854 signatures were identified, with an author-per-paper index of 7.05. Five separate co-authorship networks were built through the identification and analysis of the 4,568 author nodes and 32,647 co-authorship edges (see Table 1 for network analysis terminology). These publications were published in 312 different scientific journals. A cumulative 50% of the publications were published in 28 different journals. The remaining publications were scattered throughout 284 journals, with 190 journals publishing only one paper on the five diseases during the study period (2002–2012).

### Institutional research collaborations within Germany and abroad

Findings on institutional work and collaboration at the individual disease level are listed in Table 4, and more detailed information can be obtained in the S1 Table.

**Table 4:** Top 10 research institutions and Top 10 countries contributing to the German NTD research networks, as ranked per disease by the number of signatures (%) extracted from each publication.

Disease	Top 10 Research *Organisations*, by number of publication signatures (%)		Top 10 Research *Countries*, by number of publication signatures (%)	
**Leishmaniasis**	Charité Universitätsmedizin Berlin	224 (7.4)	**Germany**	**1462 (48.6)**
(n = 3010 signatures, incl. 491 institutions & 64 countries)	Bernhard Nocht Institute for Tropical Medicine	99 (3.3)	United Kingdom	190 (6.3)
	Julius Maximilians University of Wuerzburg	96 (3.2)	United States	177 (5.9)
	Johannes Gutenberg University Mainz	96 (3.2)	Israel	149 (5.0)
	Friedrich-Alexander-University Erlangen-Nuremberg	82 (2.7)	Brazil	89 (3.0)
	Hebrew University of Jerusalem*	69 (2.3)	France	86 (2.9)
	Ludwig Maximilians University of Munich	58 (1.9)	India	82 (2.7)
	Al-Quds University*	56 (1.9)	Belgium	68 (2.3)
	University of Muenster	55 (1.8)	Australia	62 (2.1)
	Wellcome Trust Sanger Institute*	49 (1.6)	Switzerland	57 (1.9)
**Schistosomiasis**	Ruprecht Karls University Heidelberg	56 (4.9)	**Germany**	**479 (42.1)**
(n = 1138 signatures, incl. 257 institutions & 51 countries)	Leiden University Medical Center*	52 (4.6)	United States	91 (8.0)
	Rostock University	44 (3.9)	United Kingdom	80 (7.0)
	Justus Liebig University Giessen	42 (3.7)	Netherlands	78 (6.9)
	Heinrich-Heine-University Duesseldorf	38 (3.3)	France	54 (4.8)
	Eberhard Karls University of Tuebingen	35 (3.1)	Egypt	39 (3.4)
	Ludwig Maximilians University of Munich	26 (2.3)	China	30 (2.6)
	Albert Schweitzer Hospital Medical Research Unit*	25 (2.2)	Gabon	25 (2.2)
	Friedrich-Alexander-University Erlangen-Nuremberg	22 (1.9)	Italy	24 (2.1)
	Institut Pasteur de Lille*	16 (1.4)	Switzerland	21 (1.9)
**Chagas Disease**	Charité Universitätsmedizin Berlin	55 (5.2)	**Germany**	**506 (47.8)**
(n = 1 059 signatures, incl. 220 institutions & 32 countries)	Julius Maximilians University of Wuerzburg	48 (4.5)	Brazil	161 (15.2)
	Bernhard Nocht Institute for Tropical Medicine	43 (4.1)	Argentina	86 (8.1)
	Ruprecht Karls University Heidelberg	37 (3.5)	United States	60 (5.7)
	Swiss Tropical and Public Health Institute*	25 (2.4)	United Kingdom	49 (4.6)
	University of São Paulo*	21 (2.0)	Switzerland	39 (3.7)
	University of Muenster	21 (2.0)	France	17 (1.6)
	Universidad Nacional de Rosario*	21 (2.0)	Uruguay	14 (1.3)
	Justus Liebig University Giessen	21 (2.0)	Belgium	13 (1.2)
	Federal University of Minas Gerais*	21 (2.0)	Bolivia	11 (1.0)
**Sleeping Sicknes**s	Ruprecht Karls University Heidelberg	98 (11.8)	**Germany**	**450 (54.4)**
(n = 828 signatures, incl. 180 institutions & 31countries)	Julius Maximilians University of Wuerzburg	53 (6.4)	United Kingdom	62 (7.5)
	Swiss Tropical and Public Health Institute*	31 (3.7)	Switzerland	57 (6.9)
	Free University of Berlin	27 (3.3)	Brazil	41 (5.0)
	Eberhard Karls University of Tuebingen	24 (2.9)	United States	34 (4.1)
	Ludwig Maximilians University of Munich	23 (2.8)	France	23 (2.8)
	Medical Mission Clinic Wuerzburg	17 (2.1)	Belgium	21 (2.6)
	Federal University of São Paulo*	15 (1.8)	Netherlands	20 (2.4)
	Sandler Center University of California*	15 (1.8)	Kenya	18 (2.2)
	Université Victor Segalen Bordeaux 2*	14 (1.7)	Japan	14 (1.7)
**Onchocerciasis**	Bernhard Nocht Institute for Tropical Medicine	163 (19.9)	**Germany**	**439 (53.6)**
(n = 819 signatures, incl. 134 institutions & 26 countries)	University of Bonn	78 (9.5)	United States	90 (11.0)
	Eberhard Karls University of Tuebingen	66 (8.1)	United Kingdom	84 (10.3)
	Liverpool School of Tropical Medicine*	32 (3.9)	Ghana	50 (6.1)
	Kumasi Center for Collaborative Research (KCCR) *	26 (3.2)	Cameroon	36 (4.4)
	J. Craig Venter Institute*	25 (3.1)	Tanzania	17 (2.1)
	Kwame Nkrumah University*	18 (2.2)	Canada	16 (2.0)
	University of Muenster	17 (2.1)	Italy	14 (1.7)
	New England BioLabs Inc. *	15 (1.8)	France	12 (1.5)
	Biotica Technology Limited*	11 (1.3)	Austria	11 (1.3)

* Institutions outside of Germany are written in italics.

The number of collaborating institutions and their distribution across countries varied for the different disease networks. A map of the collaboration patterns for the five diseases is presented in Fig 3. The United States and the United Kingdom were the only countries in the top five collaborating countries for all five diseases. Over 60 percent of the contributions from outside of Germany came from countries classified as high-income countries (HIC) by the Organisation for Economic Co-operation and Development (OECD) for all diseases but Chagas disease, for which the contribution was 47 percent (S2 Table). The network around Chagas disease had the highest percentage of contributions from research institutions outside of Germany coming from upper-middle-income countries (49.7%). Among the emerging economies of Brazil, Russia, India, China and South Africa (BRICS) affected by NTDs, Brazil clearly outperformed China and South Africa, with both contributing to only approximately one-tenth of the collaborations as Brazil. India is a country with high Leishmaniasis prevalence [30] and it was ranked seventh for Leishmaniasis research partners, with almost no other published research collaborations for any of the other diseases analysed here. Co-authorship collaborations with partners in low-income countries (LIC), which carry the majority of the NTD burden, were overall only a tenth of those with partners from HICs. Among the LICs, co-authorships were spread across 27 countries.

When looking at continents, not income groups, the Onchocerciasis network was the only research network in which researchers from Africa contributed more than researchers from any other continent. It was also the only disease network in which African countries were among the top five research countries. Across all the diseases, co-authorships between researchers on the African continent and Germany showed that research collaborations were spread across 11 countries; however, few of these collaborations were very active.

Among the five diseases analysed here, the German research network was spread most widely, and it was the most international for Leishmaniasis (491 institutions, 64 countries) and the least for Onchocerciasis (134 institutions, 26 countries). In those German research networks, the contributions from German research institutions themselves amount to approximately half of the overall contributions for the five diseases in focus.

For Germany, 220 different research institutions were identified as author affiliations in publications on the five NTDs under study. Ranking individual research institutions by the number of publications in which they were named as affiliations uncovered variations between the different NTDs. Among the top five German research institutions for each of the diseases, there were 12 different institutions. The Charité—Universitätsmedizin Berlin was the most frequently quoted affiliation for Chagas disease and Leishmaniasis research, whereas the Ruprecht-Karls-University of Heidelberg was the most quoted affiliation for Schistosomiasis and Sleeping Sickness research. The Bernhard-Nocht-Institute for Tropical Medicine in Hamburg was the most quoted affiliation for Onchocerciasis and the most quoted affiliation overall. Outside of Germany, 630 different research institutions were identified as affiliations in publications that had at least one co-author affiliated with a German research institution. Non-German research institutions that have frequently collaborated with German research institutions were also found among the most frequently named affiliations in the German NTD research network (Table 4).

### Social network analysis of authors in German NTD research networks

Individual researchers were identified by their number of co-authored publications, h-index and betweenness centrality (Table 5). For each disease network, the comparison of authors by either their number of publications or h-index revealed a similar top five ranking. Author results for betweenness centrality (see Table 5) also appeared to be related to the traditional indicators, as mentioned above, for the majority of leading authors in each disease network. This finding allowed us to identify leading researchers in each research field. However, betweenness centrality also enabled the identification of authors who would not have been identified with traditional indicators. For example, 13 of the 25 authors among the top five for betweenness centrality were not among the top five for any of the traditional ranking parameters. The number of authors who contributed to each disease-based co-authorship network ranged from 446 (Onchocerciasis) to 1904 (Leishmaniasis).

**Table 5:** Top 5 authors ranked by their number of publications, specific h-Index, betweenness centrality per disease network, including network parameters per disease network.

Disease	Nodes	Edges	Graph Density	Components	Giant component authors	Average Degree	Authors by	Authors by	Authors by
	(Authors)				(% of all authors)	(Maximum)	(No of publications)	(specific h-index)	(betweenness centrality *)
**Leishmaniasis**	1 904	16 200	0.09	71	1535 (80.6)	17.02 (224)	Schoenian, G (60)	Schoenian, G (22)	Schoenian, G (21 594)
							Kuhls, K (26)	Kuhls, K (13)	Anders, G (11 862)
							Bogdan, C (23)	Bogdan, C (13)	Yardley, V (10 067)
							von Stebut, E (23)	von Stebut, E (11)	Brun, R (9 004)
							Moll, H (127)	Sindermann, H (11)	Bogdan, C (8 838)
**Schistosomiasis**	858	5 632	0.02	50	495 (57.7)	13.12 (69)	Ruppel, A (17)	Grevelding, CG (9)	Doenhoff, MJ (1 679)
							Grevelding, CG (13)	Ruppel, A (8)	Grevelding, CG (1 211)
							Kremsner, PG (11)	Doenhoff, MJ (7)	Ruppel, A (961)
							Doenhoff, MJ (9)	Kremsner, PG (6)	Grobusch, MP (729)
							Richter, J (9)	Geyer, R (5)	Richter, J (709)
**Chagas Disease**	764	3 464	0.01	67	189 (24.7)	9.07 (67)	Brun, R (15)	Brun, R (8)	Brun, R (894)
							Krauth-Siegel, RL (11)	Krauth-Siegel, RL (8)	Krauth-Siegel, RL (613)
							Kaiser, M (8)	Fleischer, B (7)	Hernandez, P (384)
							Fleischer, B (8)	Kaiser, M (6)	Luquetti, AO (343)
							Heringer-Walther, S (8)s	Jacobs, T (6)	Lopes, NP (159)
**Sleeping Sickness**	596	2 911	0.02	38	315 (52.9)	9.77 (72)	Brun, R (16)	Krauth-Siegel, RL (12)	Brun, R (2 250)
							Krauth-Siegel, RL (14)	Brun, R (9)	Stich, A (1 132)
							Stich, A (11)a	Stich, A (9)	Clayton, CE (918)
							Kaiser, M (10)	Steverding, D (7)	Engstler, M (767)
							Khalid, SA (7)	Kaiser, M (6)	Kaiser, M (635)
**Onchocerciasis**	446	4 440	0.05	18	352 (78.9)	19.91 (103)	Buettner, DW (34)	Buettner, DW (15)	Buettner, DW (2 582)
							Hoerauf, A (32)	Hoerauf, A (15)	Brattig, NW (853)
							Brattig, NW (22)	Brattig, NW (11)	Hoerauf, A (812)
							Krueger, A (12)	Adjei, O (10)	Pfarr, KM (801)
							Mand, S (12)	Mand, S (9)	Koenig, R (648)

* (for authors with >2 publications because of the different sizes of the network, absolute numbers of betweenness centralities are not comparable between networks but only within the disease network itself)

The co-authorship networks organised by disease differ in terms of their density, number of components, share of authors who were part of the giant components and average degree (for an explanation of social network analysis terminology, see Table 1). In general, the Onchocerciasis and Leishmaniasis networks displayed similar characteristics, despite the former being much smaller. By contrast, the Chagas network displayed quite different network characteristics; for example, the Leishmaniasis network was the most dense (Density 0.09) and the Chagas disease network (0.01) was the least dense (Table 5). Similarly, the number of individual network components that are not connected to each other range from 18 (Onchocerciasis) to 67 (Chagas disease).

**Fig 4:**
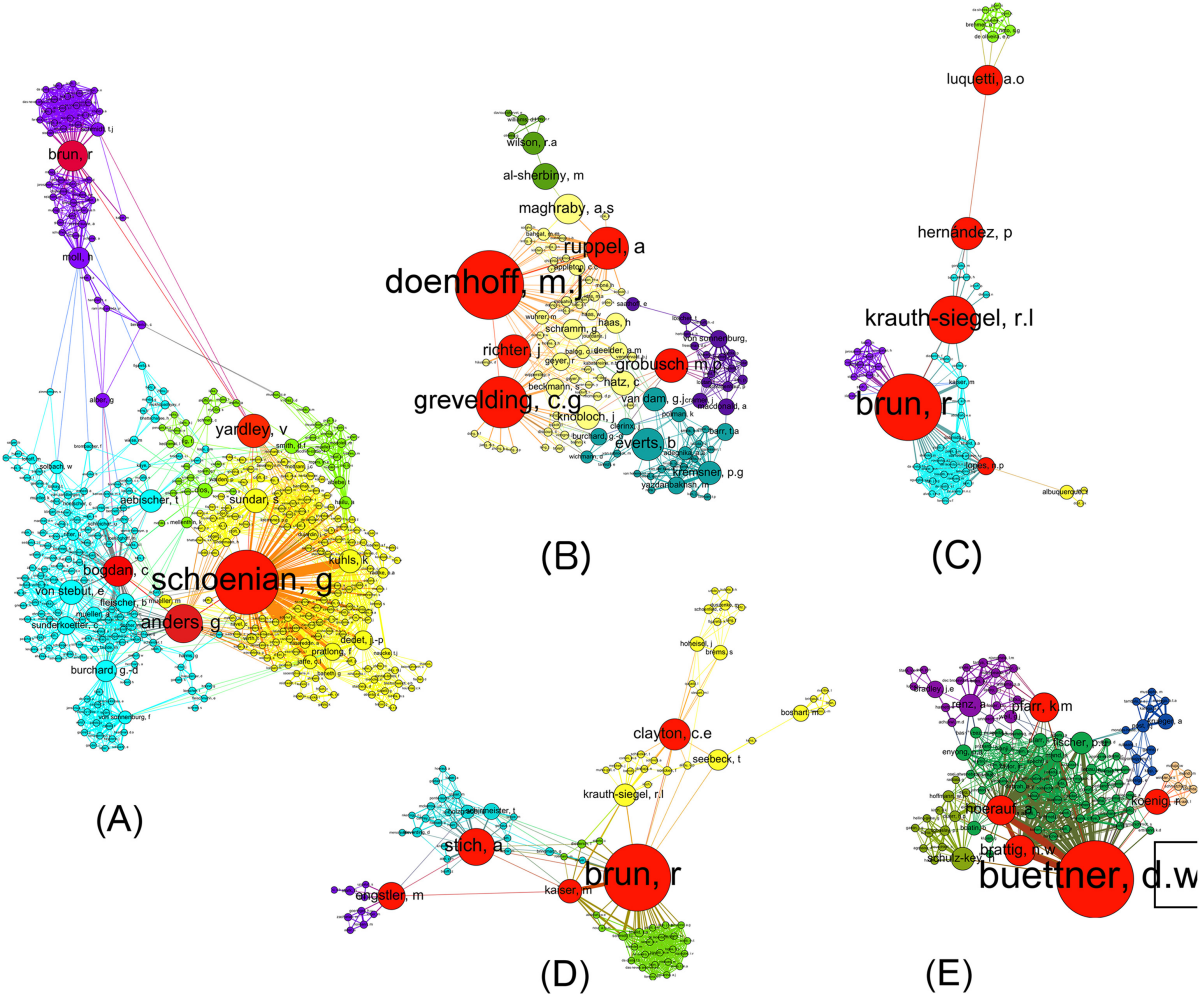
Giant components of individual co-author networks. Individual researcher networks are based on co-author networks affiliated with Germany for (A) Leishmaniasis, (B) Schistosomiasis, (C) Chagas disease, (D) Sleeping Sickness and (E) Onchocerciasis. The figure shows giant components, i.e., the components in the network that include the largest number of authors, and smaller components are not shown. The node size is scaled by betweenness centrality, and each node represents individual authors with more than two publications. Links between the nodes (edges) represent a co-authored publication. The 'Force Atlas' layout simulates repulsion forces between nodes, and thus the network spreads as far as the edges holding them together will allow, allowing for the interpretation of how closely authors are working together. For further explanation of network analysis terms, please see Table 1.

**Fig 5:**
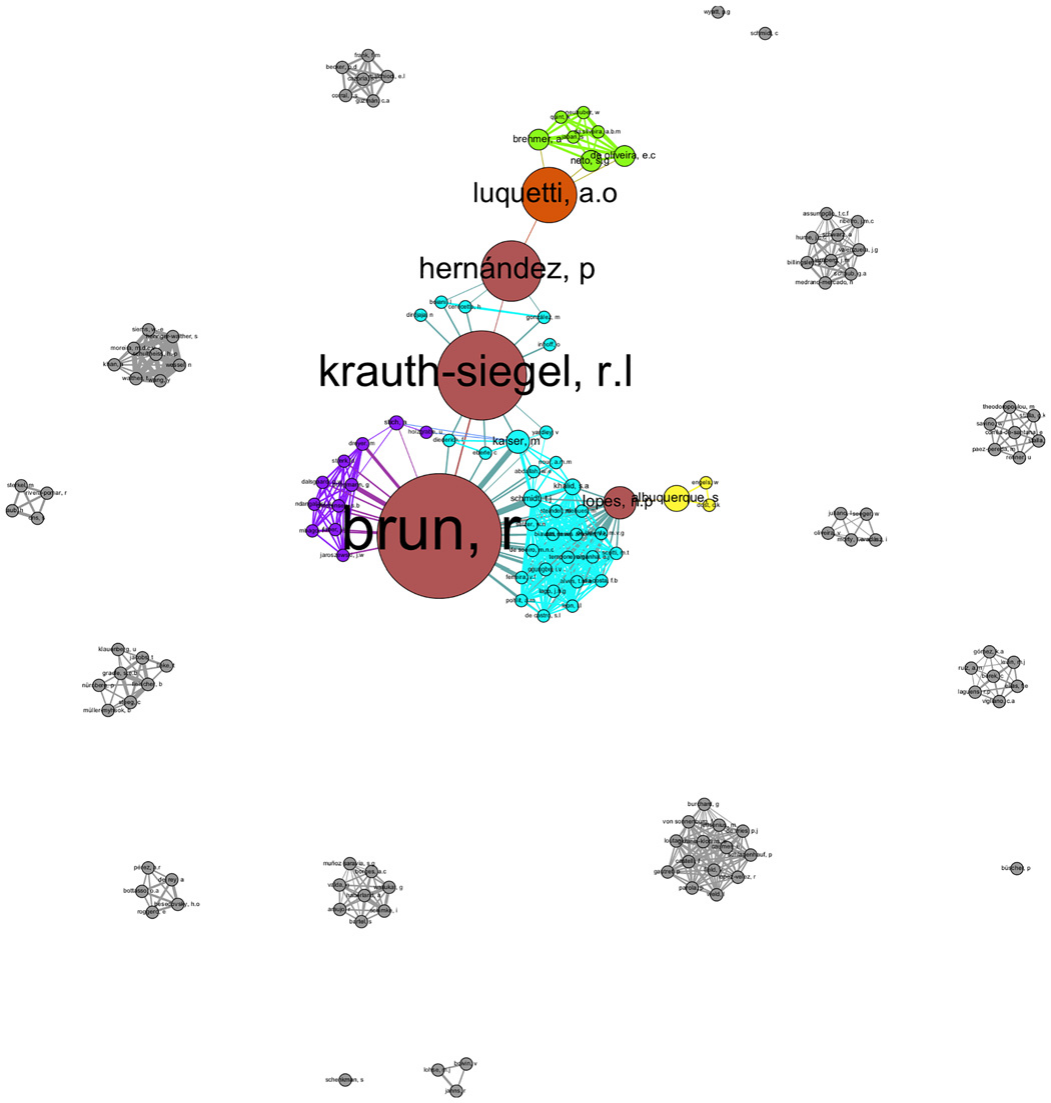
All components of the individual co-author network for Chagas disease. Giant components for Chagas disease, including all other, smaller components of the co-author network. The sizes of the nodes are scaled by betweenness centrality, and the nodes represent individual authors with more than two publications. Links between the nodes (edges) represent a co-authored publication.

We also identified one giant component for each of the networks (Fig 4). Each network’s giant component represented a varying number of authors ranging from 24.7% (Chagas disease) to 80.6% (Leishmaniasis) of all the authors in each network. For Chagas disease, this finding led to a large proportion of authors not being included in the giant component (Fig 5), but instead they were scattered among a large number of components. By contrast, the Leishmaniasis network included a similar number of components as the Chagas network despite the fact that the former included twice as many authors but a far higher proportion of authors were represented by the giant component (Table 5).

Likewise, both the Onchocerciasis and Leishmaniasis networks had a high average degree (17.0 and 19.9, respectively), which indicated that the authors in these networks have published with a large number of co-authors. This finding contrasted with the low average degree of the Chagas disease network (9.07), which indicated that those authors published with fewer co-authors on Chagas disease.

## Discussion

### Main findings

We found that until the present, the NTD research share of publications with German affiliations was considerably lower relative to medical research in general. Hence, it appears that German NTD research is lagging behind the nation’s role in other fields of health research. For research on the 11 diseases included in our study, 4.4% of global NTD publications were affiliated with Germany. This percentage is less than half the share of German research output (9%) for all research publications in the medical field [31]. Regarding NTD research specifically, Germany is not performing as strongly as other countries that show similar characteristics in other fields of research, such as the United Kingdom or France [10]. This lack of comparative strength for Germany has already been acknowledged by the German government in their recent health research framework program [32].

An analysis of co-authorship patterns with research partners abroad revealed that the majority of all contributions come from HIC, and they are dominated by the United Kingdom and the United States; the next most prolific collaborating country was Brazil. The total number of co-authorship collaborations with partners in LICs is 10 times lower than it is for HICs. The research landscape in Germany does not have clearly dominant NTD research institutions but instead spreads its endeavours across different individual universities and research units.

### Analysing institutional collaboration patterns and research capacity

Research cooperation with low- and middle-income countries has been the focus of the German government by facilitating collaboration and capacity strengthening through their support for initiatives such as the European Clinical Trial Development Partnership (EDCTP), and yet they have not been systematically analysed nor specifically analysed with social network analysis tools. Our analysis of German NTD researcher co-authorship patterns revealed that publication output remains dominated by the global north, and it is particularly dominated by collaborations of researchers from well-known research powerhouses such as the United States and the United Kingdom.

The middle-income country with the most productive collaborating researchers was Brazil, with more co-authorships than all other BRICS countries combined, and except for Onchocerciasis research, Brazilian researchers performed strongly in all other diseases analysed here. This finding reflects the Brazilian government’s strategic commitment to support NTD research [27], and it is even more remarkable considering that not all of the five diseases on which we focused here have high prevalence in Brazil [33].

Overall, the total number of co-authorships from German research partners in low-income countries was meagre, being only a tenth of the number of those from high-income countries. Analysing co-authorships between researchers on the African continent and Germany specifically showed that there are only a few very active collaborations. For all diseases, the ratio between the numbers of collaborations from the continent per number of contributing countries is lower for Africa than for Europe, North America and South America, which indicates that collaborations among high- and middle-income countries focus on a few key research partners, and collaborations with researchers from the African continent were more dispersed.

This finding suggests that capacities for research collaborations exist within a range of different countries, even though the overall number of contributions (as with signatures for co-authorships) from low-income countries remained comparatively low. The analysed publications showed collaborations between many authors within high-income countries and only a few with single authors or institutions in low-income countries. Other findings showed that among publications with African co-authors, the largest number of authors still came from countries in the global north [34]. Our data suggest that co-authorship analysis could further help identify targets for much-needed research capacity strengthening [35] and spur research productivity through diversification via collaboration [36]. In the German context, these findings are particularly pertinent because they could drive policy making for research capacity strengthening through programs that are already in place, such as the Research Networks for Health Innovations in the Sub-Saharan Africa Initiative. [18]

As expected, almost all of the publications were written in collaboration between several co-authors, with the research being published in a broad array of journals and PLoS NTD leading, though it was only launched during the study period.

### Mapping the German NTD research landscape

When analysing the NTD publication output at the disease level, even though the actual publication output in Germany is lower compared to other high-income countries, the focus on Leishmaniasis, Schistosomiasis and Chagas disease research in Germany is similar to that of international NTD research. However, the relatively higher publication output on Sleeping Sickness and Onchocerciasis is at a fifth for authors with an affiliation to Germany compared with a tenth internationally, which could indicate comparatively strong research expertise in Germany that is worthy of additional support.

Our analysis of the German NTD research landscape revealed that no single research institution in Germany is dominating or leading NTD R&D, but the research is spread across different individual universities and publicly funded research entities. This finding differs from NTD research in other high-income countries, where research efforts are more concentrated within fewer institutions. Although the publicly funded Bernhard-Nocht-Institute for Tropical Medicine in Hamburg showed the highest number of co-authorships, it is closely followed by a number of universities with research foci on different NTDs. This finding likely reflects the federal system in Germany with its rather broad approach to university-based research and education compared with the tradition of more centralised structures that exist in other countries. In addition to the relatively small amount of NTD research in Germany, this fragmentation adds to the rather low international visibility of German global health research [19,37].

### Co-authorship network analysis helps to identify academic talent and opportunities for collaboration

In contrast to the institutional environment, an analysis on the level of individual researchers suggested that German NTD R&D network hubs were dominated by a few individuals. This finding was substantiated by the fact that some leading authors were ranked highly in terms of both traditional bibliometric indicators and betweenness centrality. When analysing co-author research networks, which include German researchers and their partners abroad, we found that among the top researchers identified through traditional bibliometric indicators such as the number of publications or h-index, one (Schönian G) is an emeritus researcher and one (Büttner D) has already passed away [38]. Brun R, despite being a collaborator who works at the Swiss Tropical and Public Health Institute, has contributed substantially enough to the German NTD research network, making him the leading researcher for both Sleeping Sickness and Chagas disease.

It appears that a considerable amount of NTD R&D expertise is held among German NTD researchers approaching retirement age, and therefore the field is at risk for capacity and expertise loss. This finding emphasizes the need for a knowledge transfer to a younger generation of researchers.

It has been suggested that applying social network analysis to research, and using betweenness centrality in particular, supports the identification of researchers who are most likely to produce a higher h-index in the future, through the analysis of today’s research network structure [39]. Through the identification of individual researchers within the networks that already show a high betweenness centrality, and not yet having established a high number of publications or a high h-index, social network analysis could facilitate the identification and hence the targeting of support for younger, well-connected researchers that have not yet accumulated the years of experience and publications that bias traditional indicators towards older academics.

Stratifying the German NTD research landscape by disease allowed the identification of noticeable differences in co-authorship networks for the different diseases. These differences highlighted specific expertise and the most productive research collaborations, which might be worthy of particular support. For example, the Leishmaniasis and Onchocerciasis networks were characterized by a large number of collaborations between authors, as indicated by a high average degree and network density. Although this finding was no surprise with regards to Leishmaniasis, which was by far the most researched disease of those analysed here, it was more remarkable for Onchocerciasis, supporting the evidence that this disease could be a comparative strength in German NTD research. Conversely, co-authorship network analysis allowed the identification of ‘gaps’ or missed opportunities, e.g., the Chagas disease research network showed great potential because of its strong contributions from middle-income countries such as Brazil, but the network remained scattered among many components at a low density, which indicates that there is room for improved collaborations between the actors who are already involved.

It is of further concern to us that the potential looming public sector NTD R&D capacity loss among aging researchers in Germany is mirrored by the current near-absence of infectious disease R&D capacities within German pharmaceutical companies, which were once global leaders in infectious and neglected tropical disease research (a fact that was even used as propaganda under dubious circumstances [40]).

### Limitations

We only used data from the SCOPUS bibliometric database, which was found to have the widest coverage of NTD literature [41]. Future research network analyses should consider the exploration of other literature databases, for example, Web of Science and MEDLINE, to identify additional publications.

Not including agent names in our search string may have limited the number of hits related to basic research studies; however, we wanted to focus our search strategy on disease names to consider the most relevant outcomes from the perspective of patients who were affected by NTDs.

Although network analysis tools are manifold, it is important to note that we use quantifiable data such as publication output as the best available proxy measure for researcher knowledge or expertise. Additionally, the value of information gained here can only be as good as the data that is available for comparison. We considered this not only a limitation but also a call for further investigation into the structure of other NTD research networks around the world. Because our method is based solely on open source software, it can easily be reproduced in other contexts and might help to put our findings into a broader perspective.

### International policy implications

Current German government policy clearly pursues an increasing role in global health, and recent studies have acknowledged a growth in German public sector funding [22], even labeling Germany as an ‘emerging leader’, though its own funding program for NTD research expired at the end of 2014 and a renewed call for proposals is pending for 2015. It remains to be seen if and how the German government's political will, as expressed for example by putting neglected and poverty-related diseases on the agenda for the G7 Summit in Germany in June 2015 [42], is going to be reflected in measurable research output from Germany. As it hands over the G7 presidency to Japan, we urge the German Government to make good on the promises made in the G7 leaders' declaration [43].

Our findings underline the G7 national academies' of science call for policy changes [44], ‬‬‬‬particularly for promoting research collaborations and technology transfer in LMIC and to intensify research within the G7 countries themselves.

Providing process and output-based insights in NTD R&D, such as those provided here, will have an important role in the realisation of the G7 goals and for WHO’s Global Health Research and Development Observatory [45].

### Conclusions

The first systematic assessment of the German health and medical research landscape for NTD using authorship networks based on bibliometric metadata demonstrated not only the potential of social network analysis as a tool to apply to the R&D field, but it also revealed valuable findings when used to assess German research capacities in selected NTDs. Our findings showed that 4.4% of all NTD publications worldwide involve an author from a German research institution. This rather low output of German R&D activities on NTD is scattered across numerous publicly funded research institutions without single outstanding centres. Most publications that included researchers from Germany were related to other high-income countries and the emerging economy in Brazil.

Our results could contribute to identify research strengths that can be enhanced, e.g., by expanding targeted collaborations for research capacity building in LMIC, or for weaknesses to amend, for example, through encouraging collaboration in areas of shared expertise that were missed until now.

Future research should provide further in-depth analysis of individual researcher and network productivity, scientific impact and translational success in the development of new products for NTDs. Similar analyses could also include qualitative approaches (e.g., focus groups or semi-structured interviews) with key researchers and policy makers to identify barriers, e.g., limiting factors for collaboration with partners in low- and middle-income countries.

Notwithstanding the apparent political will of the current German government, our network analysis shows that NTD R&D in Germany is scattered and at risk of expertise loss. For a renewed German NTD research-funding program, it appears to be crucial to analyse the existing R&D landscape empirically to inform future research funding decisions. This analysis could be strengthened through innovative tools such as network analysis. Mapping research collaborations with partners abroad can support decisions on the selective strengthening of research capacity. Furthermore, a social network analysis could provide valuable insights into which specific diseases could be prioritised based on where comparative advantages in research networks are found. This direction is essential for developing a data-driven research strategy to expand Germany’s research activities in the field of NTDs.

## Supporting Information

S1 Dataset Chagas disease author network dataset. (GEXF)

S2 Dataset Chagas disease institutional network dataset with GPS data. (GEXF)

S3 Dataset Sleeping sickness author network dataset. (GEXF)

S4 Dataset Sleeping Sickness institutional network dataset with GPS data. (GEXF)

S5 Dataset Leishmaniasis author network dataset. (GEXF)

S6 Dataset Leishmaniasis institutional network dataset with GPS data. (GEXF)

S7 Dataset Onchocerciasis author network dataset. (GEXF)

S8 Dataset Onchocerciasis institutional network dataset with GPS data. (GEXF)

S9 Dataset Schistosomiasis author network dataset. (GEXF)

S10 Dataset Schistosomiasis institutional network dataset with GPS data. (GEXF)

S1 Table All affiliated research institutions and countries in the global German NTD research network, as ranked by occurrence number for affiliation in the publications for each disease. (XLSX)

S2 Table Co-authorships in the global German NTD Research network by continent and OECD income group, counted by occurrence number by the country name for affiliation in the publications for each disease. (DOCX)
