# Supplementary material for: Using Co-authorship Networks to Map and Analyse Global Neglected Tropical Disease Research with an Affiliation to Germany
Source: PLoS Negl Trop Dis. 2015 Dec 31;9(12):e0004182. doi: 10.1371/journal.pntd.0004182 (PMC4703140; doi:10.1371/journal.pntd.0004182)
Supplement: S2 Table — (DOCX) [file pntd.0004182.s012.docx]

| **DISEASE** | **Affiliations By Continent** | | **Affiliated Countries On That Continent** | **Affiliations By Income Group (Oecd)** | | **Affiliated Countries In That Group** | |  |
| --- | --- | --- | --- | --- | --- | --- | --- | --- |
| LEISHMANIASIS | Africa | 113 | 13 | Low income | 69 | | 7 | |
|  | Asia | 361 | 18 | Lower middle income | 133 | | 13 | |
|  | Australia | 69 | 2 | Upper middle income | 239 | | 16 | |
|  | Europe (without Germany) | 628 | 17 | High income: OECD (without Germany) | 1096 | | 24 | |
|  | North America | 220 | 4 | High income: nonOECD | 3 | | 2 | |
|  | South America | 157 | 9 |  |  | |  | |
| SCHISTOSOMIASIS | Africa | 135 | 18 | Low income | 41 | | 10 | |
|  | Asia | 70 | 12 | Lower middle income | 58 | | 7 | |
|  | Australia | 14 | 1 | Upper middle income | 92 | | 8 | |
|  | Europe (without Germany) | 323 | 15 | High income: OECD (without Germany) | 449 | | 21 | |
|  | North America | 94 | 2 | High income: nonOECD | 19 | | 4 | |
|  | South America | 23 | 2 |  |  | |  | |
| CHAGAS | Africa | 24 | 5 | Low income | 16 | | 3 | |
|  | Asia | 13 | 4 | Lower middle income | 20 | | 4 | |
|  | Australia | 0 | 0 | Upper middle income | 255 | | 6 | |
|  | Europe (without Germany) | 162 | 12 | High income: OECD (without Germany) | 247 | | 16 | |
|  | North America | 66 | 3 | High income: nonOECD | 15 | | 2 | |
|  | South America | 288 | 7 |  |  | |  | |
| HAT | Africa | 72 | 14 | Low income | 46 | | 9 | |
|  | Asia | 19 | 3 | Lower middle income | 16 | | 4 | |
|  | Australia | 0 | 0 | Upper middle income | 55 | | 5 | |
|  | Europe (without Germany) | 209 | 9 | High income: OECD (without Germany) | 261 | | 12 | |
|  | North America | 34 | 1 | High income: nonOECD | 0 | | 0 | |
|  | South America | 44 | 3 |  |  | |  | |
| ONCHOCERCIASIS | Africa | 139 | 11 | Low income | 49 | | 7 | |
|  | Asia | 3 | 2 | Lower middle income | 89 | | 4 | |
|  | Australia | 4 | 1 | Upper middle income | 2 | | 1 | |
|  | Europe (without Germany) | 126 | 8 | High income: OECD (without Germany) | 238 | | 12 | |
|  | North America | 106 | 2 | High income: nonOECD | 2 | | 1 | |
|  | South America | 2 | 1 |  |  | |  | |
